# A preliminary study of bowel rest strategy in the management of *Clostridioides difficile* infection

**DOI:** 10.1038/s41598-020-79211-3

**Published:** 2020-12-16

**Authors:** Hiroshi Sugimoto, Ayaka Yoshihara, Takao Yamamoto, Keisuke Sugimoto

**Affiliations:** 1 Department of Respiratory Medicine, Kobe Red Cross Hospital, 1-3-1 Wakinohama Kaigan-dori, Chuo-ku, Kobe, 651-0073 Japan; 2 Department of Gastroenterology, Kobe Red Cross Hospital, Kobe, Japan

**Keywords:** Outcomes research, Diarrhoea, Clostridium difficile

## Abstract

*Clostridioides difficile* infection (CDI) is an important nosocomial infection and is the leading cause of infectious diarrhea in hospitalized patients. We aimed to assess the effect of bowel rest on the management of CDI. A single-center retrospective cohort study was conducted. The primary outcome was the composite of the all-cause mortality and CDI recurrence within 30 days. The main secondary outcome was switching from metronidazole to vancomycin. Of the 91 patients with CDI enrolled as the full cohort, 63 patients (69%) and 28 patients (31%) constituted the control group and the bowel rest group, respectively. After one-to-one propensity score matching, a total of 46 patients were included as the matched cohort. In the full cohort, the composite outcome occurred in 19.0% and 14.3% of the patients in the control and the bowel rest group, respectively (p = 0.768). In the matched cohort, it was 17.4% in each group. Although there was no statistically significant difference, the trend of switching was lower in the bowel rest group. The bowel rest may not affect the all-cause mortality and CDI recurrence within 30 days. However, in those prescribed bowel rest, switching from metronidazole to vancomycin may reduce.

## Introduction

*Clostridioides difficile* is an anaerobe colonizing approximately 5–10% of adults and occasionally causes *C. difficile* infection (CDI) which is characterized by severe diarrhea^1^. Although the burden of CDI has recently reduced due to the efforts of healthcare workers^2^, it remains an important nosocomial infection that is the leading cause of infectious diarrhea in hospitalized patients^3^.

Clinicians often apply bowel resting strategy (fasting and fluid replacement) for the management of CDI as well as other diarrheal diseases. However, the decision of whether to put a patient with CDI on bowel rest or not remains unclear and is at the treating doctor’s discretion. The previous clinical practice guidelines on CDI empirically recommended the continued use of oral or enteral feeding, if tolerable^4^. However, the latest clinical practice guidelines on CDI provides no such dietary recommendations^5^. To the best of our knowledge, there are no previous reports that have assessed the effect of bowel rest in the management of CDI.

The intestinal microbiota provides essential health benefits to its host and bowel rest is a known contributor to changes in the gut microbiota^6^. Therefore, we hypothesized that bowel rest can affect patients with CDI. In this study, we aimed to assess the effect of bowel rest on the management of CDI in clinical settings.

## Methods

### Study design

This propensity score-matched retrospective cohort study was conducted at Kobe Red Cross Hospital in Japan, from August 2010 to February 2020. The inclusion criteria were all admitted patients ≥ 18 years of age who were clinically diagnosed with CDI based on a positive fecal toxin test (enzyme immunoassay) and administered metronidazole, vancomycin, or fidaxomicin as the initial treatment for CDI. The exclusion criteria were as follows: individuals who (1) opted to be excluded from this study by opting out on the website, (2) had a prior history of CDI within the last 3 months, or (3) had already been on bowel rest 3 days before the initial treatment for CDI. This study was reviewed and approved by the Kobe Red Cross Hospital Ethics Committee institutional review board (approval number: 199) and has been performed in accordance with the ethical standards laid down in the 1964 Declaration of Helsinki and its later amendments. Individual informed consent was waived off by the Kobe Red Cross Hospital Ethics Committee institutional review board.

### Variables

The bowel rest group comprised of patients who had discontinued diet (for at least 24 h) after the starting of the initial treatment for CDI; the rest of the patients served as the control group. The following clinical characteristics were compared between the two groups: age, sex, comorbidities (diabetes, malignancy, and inflammatory bowel disease), initial treatment for CDI (metronidazole, vancomycin, or fidaxomicin), antibiotic use (pre-treatment and post-treatment), medications [proton pump inhibitor (PPI), histamine H2-receptor antagonist (H2RA), probiotics, immunosuppressants, chemotherapy, and vasopressor], tube feeding, surgery, intensive care unit (ICU) stay, and laboratory data (serum albumin, creatinine levels, and white blood cell count). Data of all the patients were extracted from electronic medical records in our hospital.

### Outcomes

The primary outcome was the composite the all-cause mortality and CDI recurrence within 30 days. CDI recurrence was defined as the administration of metronidazole, vancomycin, or fidaxomicin for the treatment of clinically suspected CDI at least one day after the end of the initial treatment for CDI. The main secondary outcome was the percentage of patients who underwent continuous switching from metronidazole to vancomycin. The characteristics of the patients were compared between groups both before and after propensity score matching.

### Statistical analyses

To minimize selection bias in the bowel rest group, propensity score matching was done. For estimating the propensity score, a logistic regression analysis was performed using bowel rest as the independent variable. The dependent variables were age, sex, comorbidities (diabetes, malignancy, and inflammatory bowel disease), initial treatment of CDI, antibiotic use (pre-treatment), medications (PPI, H2RA, probiotics, immunosuppressants, chemotherapy, and vasopressor), tube feeding, surgery, ICU stay, and laboratory data (albumin, creatinine, and white blood cell count). Thereafter, one-to-one matching without replacement was performed using the nearest neighbor match based on estimated propensity scores of each patient with a caliper width set to 0.2 times the standard deviation of the logit of the propensity score. The C-statistics and side-by-side box plots were used to check the balancing of the matching.

Clinical characteristics and outcomes were compared between the control group and the bowel rest group and separately in the full cohort and matched cohort. To check the significance of the difference in continuous variables and categorical variables, Mann–Whitney’s U test and Fisher’s exact test were used, respectively. Statistical analyses were performed in April 2020 using R (version 4.0.0) and EZR software^7^. A p-value < 0.05 was considered statistically significant.

## Results

After the application of the inclusion and exclusion criteria, a total of 91 patients identified with CDI were recruited and they comprised the full study cohort. Among these, 63 patients (69%) were categorized into the control group and 28 patients (31%) into the bowel rest group. Metronidazole was the initial treatment for CDI in approximately 80% of the patients. Table 1 shows the clinical characteristics of the full study cohort. The two groups were comparable in all the parameters except sex which showed that the number of males was higher in the control group than the bowel rest group (p = 0.023).

**Table 1:** Clinical characteristics of the full study cohort.

Variables	Control (n = 63)	Bowel rest (n = 28)	p-value
Age (years)	84.0 (78–88)	82.5 (76–91)	0.884
Males	35 (55.6)	8 (28.6)	0.023
**Comorbidities**			
Diabetes	7 (11.1)	7 (25.0)	0.117
Malignancy	20 (31.7)	5 (17.9)	0.210
IBD	1 (1.6)	2 (7.1)	0.223
**Initial treatment for CDI**			
Metronidazole	51 (81.0)	23 (82.1)	1.000
Vancomycin	12 (19.0)	5 (17.9)	1.000
**Antibiotic use**			
Pre-treatment	55 (87.3)	23 (82.1)	0.529
Post-treatment	26 (41.3)	14 (50.0)	0.497
**Medications**			
PPI	32 (50.8)	15 (53.6)	0.824
H2RA	6 (9.5)	2 (7.1)	1.000
Probiotics	44 (69.8)	18 (64.3)	0.632
Immunosuppressants	5 (7.9)	2 (7.1)	1.000
Chemotherapy	5 (7.9)	1 (3.6)	0.662
Vasopressor	4 (6.3)	1 (3.6)	1.000
Tube feeding	9 (14.3)	5 (17.9)	0.755
Surgery	13 (20.6)	5 (17.9)	1.000
ICU stay	4 (6.3)	3 (10.7)	0.672
**Laboratory data**			
Albumin	2.30 (2.00–2.70)	2.40 (2.08–2.82)	0.475
Creatinine	0.81 (0.55–1.07)	0.72 (0.63–1.24)	0.692
WBC	8200 (5900–12,800)	10,500 (6700–14,675)	0.387

Data are median (interquartile range) or number (%).

*IBD* inflammatory bowel disease, *CDI* *Clostridioides difficile* infection, *PPI* proton pump inhibitor, *H2RA* histamine H2-receptor antagonist, *ICU* intensive care unit, *WBC* white blood cell count.

**Figure 1:**
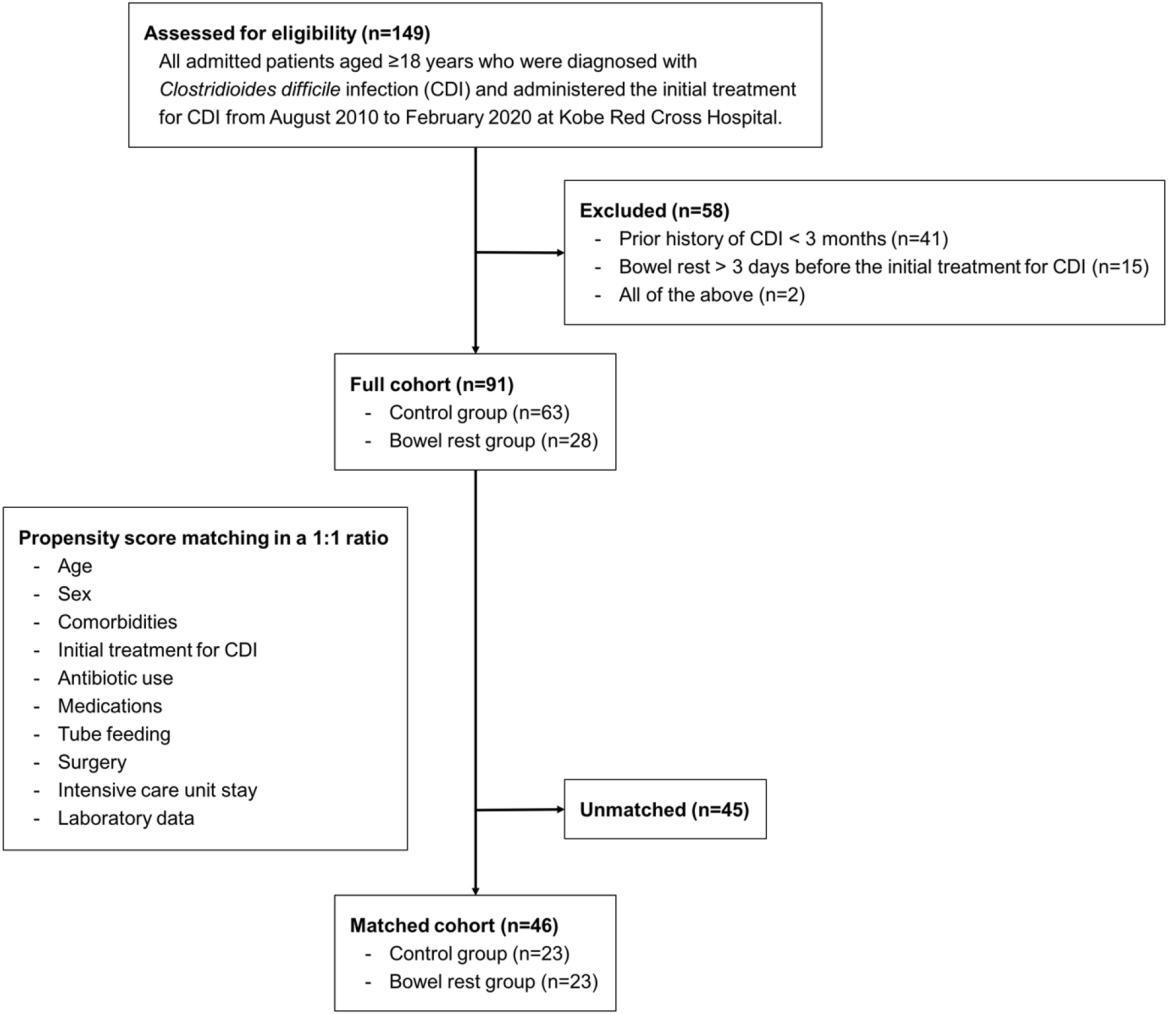
Flowchart of patients enrolled in this study.

**Figure 2:**
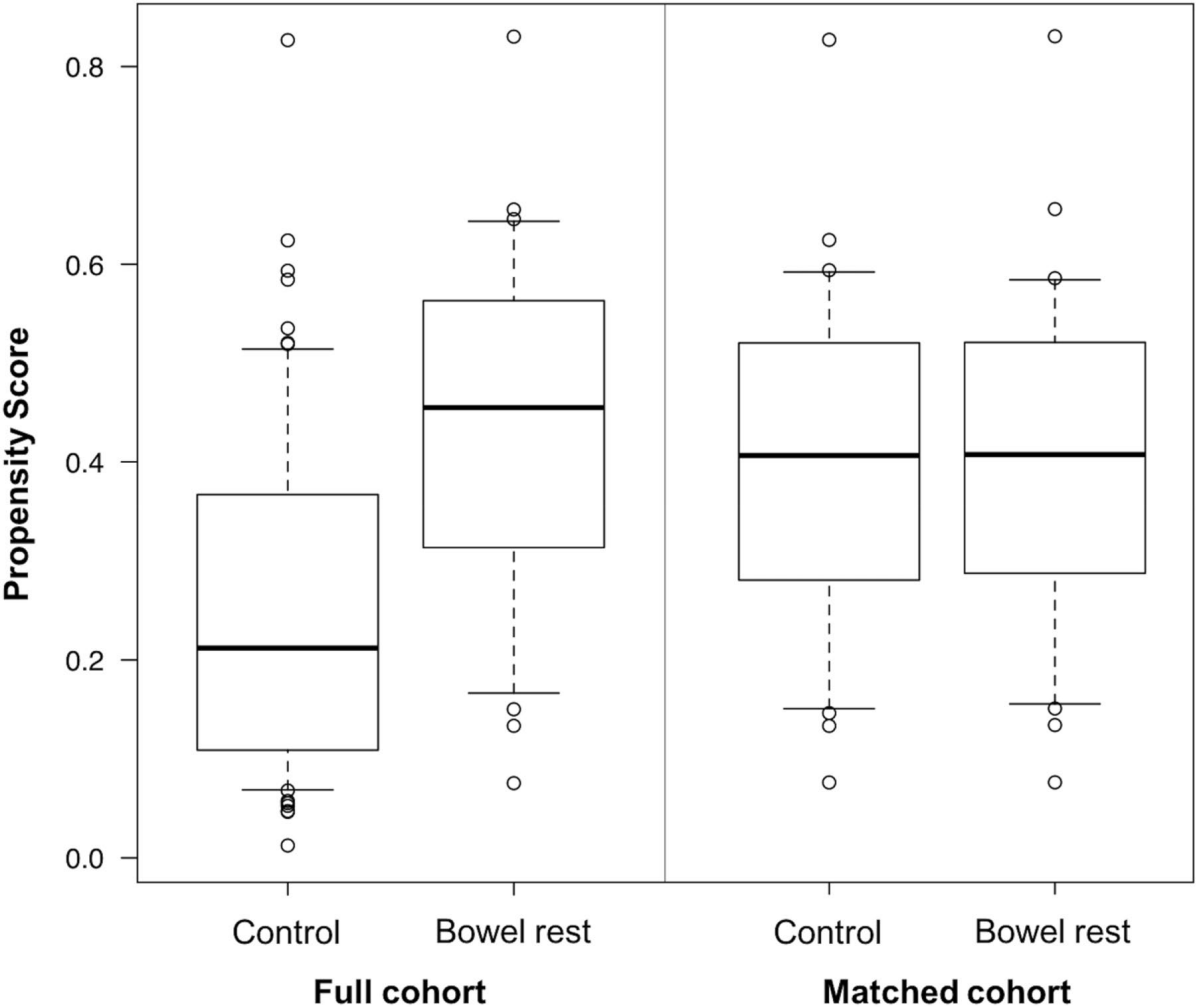
Side-by-side box plots before and after propensity score matching.

After propensity score matching (C-statistics 0.77), a total of 46 patients (23 patients in each group) were included in the matched cohort (Fig. 1). Figure 2 depicts the side-by-side box plots before and after matching. Table 2 shows the clinical characteristics in the matched cohort; there were no statistically significant differences between the groups.

**Table 2:** Clinical characteristics in the propensity score-matched cohort.

Variables	Control (n = 23)	Bowel rest (n = 23)	p-value
Age (years)	85.0 (83–88)	81.0 (77–92)	0.758
Males	5 (21.7)	7 (30.4)	0.738
**Comorbidities**			
Diabetes	6 (26.1)	5 (21.7)	1.000
Malignancy	5 (21.7)	4 (17.4)	1.000
IBD	1 (4.3)	2 (8.7)	1.000
**Initial treatment for CDI**			
Metronidazole	19 (82.6)	19 (82.6)	1.000
Vancomycin	4 (17.4)	4 (17.4)	1.000
**Antibiotic use**			
Pre-treatment	19 (82.6)	18 (78.3)	1.000
Post-treatment	9 (39.1)	11 (47.8)	0.767
**Medications**			
PPI	13 (56.5)	11 (47.8)	0.768
H2RA	1 (4.3)	2 (8.7)	1.000
Probiotics	13 (56.5)	15 (65.2)	0.763
Immunosuppressants	0 (0)	2 (8.7)	0.489
Chemotherapy	2 (8.7)	1 (4.3)	1.000
Vasopressor	1 (4.3)	1 (4.3)	1.000
Tube feeding	3 (13.0)	3 (13.0)	1.000
Surgery	5 (21.7)	4 (17.4)	1.000
ICU stay	2 (8.7)	2 (8.7)	1.000
**Laboratory data**			
Albumin	2.40 (2.10–2.75)	2.40 (2.05–2.80)	0.700
Creatinine	0.86 (0.57–1.29)	0.73 (0.66–1.25)	0.835
WBC	8200 (6450–15,000)	7600 (6600–12,550)	0.717

Data are median (interquartile range) or number (%).

*IBD*, inflammatory bowel disease; *CDI*, *Clostridioides difficile* infection; *PPI*, proton pump inhibitor; *H2RA*, histamine H2-receptor antagonist; *ICU*, intensive care unit; *WBC*, white blood cell count.

The results of outcome analyses before and after matching are shown in Table 3. In the full cohort, the composite outcome occurred in 19.0% of the patients (95% CI 10.2–30.9) in the control group and 14.3% patients (95% CI 4.0–32.7) in the bowel rest group. In the matched cohort, the composite outcome occurred in 17.4% of the patients (95% CI 5.0–38.8) in each group. Both before and after matching, there were no statistically significant differences in the primary and secondary outcomes. However, the percentage of patients being switched from metronidazole to vancomycin showed a trend to be lower in the bowel rest group than in the matched cohort [26.1% (95% CI 10.2–48.4) vs. 4.3% (95% CI 0.10–21.9)] (p = 0.096).

**Table 3:** Summary of outcomes in the study cohort.

Full cohort	Control (n = 63)	Bowel rest (n = 28)	p-value
Composite outcome within 30 days	12 (19.0 [10.2–30.9])	4 (14.3 [4.0–32.7])	0.768
All-cause mortality	9 (14.3 [6.7–25.4])	3 (10.7 [2.3–28.2])	0.749
Recurrence of CDI	3 (4.8 [1.0–13.3])	1 (3.6 [0.10–18.3])	1.000
Switching of metronidazole to vancomycin	10 (15.9 [7.9–27.3])	2 (7.1 [0.90–23.5])	0.331

Matched cohort	Control (n = 23)	Bowel rest (n = 23)	p-value
Composite outcome within 30 days	4 (17.4 [5.0–38.8])	4 (17.4 [5.0–38.8])	1.000
All-cause mortality	3 (13.0 [2.8–33.6])	3 (13.0 [2.8–33.6])	1.000
Recurrence of CDI	1 (4.3 [0.10–21.9])	1 (4.3 [0.10–21.9])	1.000
Switching of metronidazole to vancomycin	6 (26.1 [10.2–48.4])	1 (4.3 [0.10–21.9])	0.096

Data are number (% [95% confidence interval]).

*CDI*, *Clostridioides difficile* infection.

## Discussion

In the present study, we demonstrated two major clinical findings relating to bowel rest in the management of CDI. First, there were no statistically significant differences in both the composite outcome and its components (all-cause mortality or CDI recurrence) within 30 days. Second, the percentage of patients who were switched from metronidazole to vancomycin also showed no statistically significant difference between the two groups; however, it showed a lower trend in the bowel rest group in both the full cohort analysis and the propensity score-matched cohort.

The first finding that there were no statistically significant differences in the all-cause mortality or CDI recurrence within 30 days suggests that bowel rest may not affect these outcomes which is in contrast to our hypothesis. Previous studies have reported all-cause mortality of 3.4–15.1% within 30 days in patients with CDI which is similar to that reported in our study (approximately 13%)^8^. Meanwhile, the CDI recurrence rate within 30 days was approximately 4% in our study; this was within the previously reported range of 3.3–27.3%^8^. Since CDI recurrence was defined as the repetition of treatment for CDI after the initial treatment in our study, we believe that CDI recurrence may have been underestimated compared to studies where the definition of CDI was defined by the recurrence of diarrhea alone. Based on the value of C-statistics and side-by-side box plots, our propensity score matching was considered well-balanced. Although the sample size may be insufficient to conclude, our data suggests that there were no significant clinical differences in the all-cause mortality or CDI recurrence within 30 days between the control group and bowel rest group.

The second finding that the proportion of switching from metronidazole to vancomycin had a trend to be lower in the bowel rest group suggests that those on bowel rest may have a lower vancomycin use. We believe that the cause of this trend may be because diarrhea is more likely to sustain in patients continuing oral or enteral feeding. As a result, the initial treatment with metronidazole may be judged as clinically failed. Although oral metronidazole is no longer recommended as the initial treatment for CDI under the current American guideline^5^, oral metronidazole is still used as the primary regimen for CDI in Japan^9^. Unnecessary use of oral vancomycin has the potential risk of developing highly resistant bacteria such as vancomycin-resistant enterococci, although this is debatable^10^. Thus, we suggest that the use of vancomycin can be reduced by giving bowel rest instead; this would prove beneficial towards the antimicrobial stewardship.

The present study has some limitations. First, this study was a single-center retrospective study and the number of analyzed patients was small. Furthermore, we did not examine all the factors that can affect the management or outcomes; e.g., the patients’ activities of daily living, the severity of diarrhea, other comorbidities, reasons for hospitalization, changes of intestinal microbiota, and the strain of *C. difficile*. We assessed outcomes within 30 days; thus, long-term outcomes may have differed. Therefore, further well-designed investigations with a higher number of patients are warranted.

In conclusion, the bowel resting strategy may not affect the all-cause mortality and recurrence of CDI within 30 days in the management of CDI. Further, switching from metronidazole to vancomycin is possibly reduced by bowel rest.
